# Immunochemistry-based quantification of tumor-infiltrating lymphocytes and immunoscore as prognostic biomarkers in bladder cancer

**DOI:** 10.1186/s43046-024-00212-8

**Published:** 2024-03-25

**Authors:** Sarra Ben Rejeb, Sirine Elfekih, Nadia Kouki, Rami Boulma, Hassen Khouni

**Affiliations:** 1 Department of Pathology, Hopital des forces de sécurité intérieure de la Marsa, Tunis, Tunisia; 2 Department of Urology, Hopital des forces de sécurité intérieure de la Marsa, Tunis, Tunisia; 3 Faculty of Medecine of Tunis, Tunis Manar University, Tunis, Tunisia

**Keywords:** Bladder cancer, Immunoscore, Immunochemistery, Prognosis

## Abstract

**Background:**

Tumor-infiltrating lymphocytes (TILs) and the derived immunoscore (IS) have gained considerable attention over the last decade as prognostic markers in many solid cancers. However, in bladder cancer (BC), their prognostic value is not clearly established.

**Methods:**

The present study aimed to quantify the TILs rates in BC, assess the derived immunoscore, and investigate their prognostic value. An immunochemistry-based quantification of the different subtypes of TILS was performed on paraffin-embedded blocks from patients with invasive urothelial carcinoma of the bladder. We have assessed the rates of TILs, respectively, on peri-tumoral (PT) and intra-tumoral (IT) areas and calculated for each case the corresponding IS which is the index: CD8+/CD3+ TILs. The IS was then classified as low (I0, I1) or high (I2, I3, I4). We included 30 cases in the analysis.

**Results:**

The median age of patients was 65 years with a sex ratio of 9. TILs densities and distribution were significantly variable between IT and PT areas CD3+ (*p* = 0.03) and CD8+ (*p* = 0.004) with the highest rates on the PT areas. In univariate analysis, a low density of CD8+ TILs was significantly associated with an advanced age (*p* = 0.05), with the presence of lympho-vascular invasion (*p* = 0.02) and with the absence of specific histological subtype (*p* = 0.05). A low immunoscore was significantly associated with the presence of lympho-vascular invasion (*p* = 0.004). No significant association was found between TILs subpopulations, the IS, and the other clinicopathological and survival data. The overall survival (OS) and disease-free survival (DFS) medians were slightly superior in highly T (CD3+/CD8+)-cell infiltrated tumors as well as tumors with a high IS densities. However, the univariate analysis showed that TILs and immunoscore did not impact overall survival (OS) and disease-free survival (DFS).

**Conclusion:**

TILs and immunoscore might be effective prognostic tools in BC. However, standardized quantification methods and further investigation on larger samples are highly recommended to definitively attest the prognostic value of TILs and IS in BC.

## Introduction

Bladder cancer (BC) is the 9th most common malignant neoplasm in the world and the 13th leading cause of cancer death [1]. Urothelial carcinoma (UC) is the main histological subtype, accounting for > 90% of BC. Treatment protocols depend on histological prognostic factors, especially tumor stage and pathological grade [2]. Indeed, nearly 75% of BC patients present with non-muscle-invasive tumors, and the standard treatment is endoscopic resection followed by intravesical Bacillus-Calmette-Guerin (BCG) therapy. However, for patients with muscle-invasive UC and non-muscle-invasive disease with a high grade, the current standard of treatment consists on radical cystectomy (RC) with pelvic lymphadenectomy.

Unfortunately, despite improvements in surgical techniques and therapeutic approaches, the clinical outcome of patients with BC, either muscle-invasive or non-muscle invasive, remains dismal, with a high rate of relapse, progression, distant metastasis, and an overall 5-year survival rate of 15–20%.

These findings raised the need to focus on new biomarkers that could improve the prognostication of BC.

In this regard, the tumor immune microenvironment is becoming one of the most studied prognostic factors in many solid cancers, such as colorectal and breast cancer [3]. Tumor-infiltrating lymphocytes (TILs) are a component of the tumor microenvironment that has a key role in the anti-tumor immune response [4, 5]. It is well established that an increased number of immune cells correlates with a favorable clinical outcome in various malignancies, such as melanoma, colorectal, lung, and breast cancers [6, 7].

In BC, few studies have investigated the prognostic role of TILs, and their results were highly variable. Some studies have reported that an increased rate of CD3+ TILs was associated with a favorable outcome [8]; in other studies, a high rate of CD3+ and CD8+ TILs was associated with tumor relapse and progression [9]. These discrepancies may be in part justified by different methods of TILs assessment.

In this regard, the immunoscore (IS) established by IHC is considered to be a robust and reproducible tool for immune contexture assessment and quantification in solid tumors [10].

The aim of this study was to firstly assess the density of TILs in UC using IHC and to secondly investigate the prognostic role of TILs and IS in BC.

## Methods

### Study design

We have retrospectively collected cases of patients diagnosed with primary UC invading at least the connective tissue (> = pt1) diagnosed between 2011 and 2021 in our pathology department.

We have excluded from our study the following:

- Cases with non-urothelial histology
- Insufficient tumor sample or large thermal artifacts
- Patients with incomplete clinical data (follow-up)

The tumor tissue in the formalin-fixed paraffin-embedded blocks was retrieved from 22 biopsies and 13 radical cystoprostatectomy specimens.

Clinical data were retrieved from the patient’s medical records. They included the following: age, gender, tumor recurrence and progression, metastasis, treatment, survival, and time of death.

The following pathological characteristics were collected from pathology reports: tumor size, tumor focality, histological subtype, tumor grade, and the presence of in situ carcinoma, lympho-vascular invasion, lymph node metastasis, and pathologic stage.

### Immunochemistry analysis (IHC)

All tumor slides have been firstly reviewed by two pathologists to select the ones with high immune cell densities. Secondly, the corresponding paraffin-embedded blocks have been retrieved. All cases have been tested respectively, with the CD3 monoclonal antibody (Leica; LN10) and the CD8 monoclonal antibody (Leica; 4B11) available in a ready-to use form.

We have performed the immunohistochemical technique with an automated immunostainer (Leica BOND-MAX) according to the manufacturer’s protocol. The detection of immunolabeling was performed using 3-3-odiaminobenzidine ethylcarbazole chromogen and counterstained with hematoxylin. Appropriate negative and positive controls were used.

For both CD3 and CD8 antibodies, the staining was assessed as positive when it was either membranous or cytoplasmic.

To quantify, respectively, CD3+ and CD8+ positive cells, hot spot areas were selected, and the number of stain-positive cells was counted at tumor margins (TM) and intra-tumoral (IT) fields. To eliminate sampling errors, the counting was performed on five noncontiguous high-power fields (×400).

The final density was the sum of the five areas respectively in TM and IT for each antibody. As no cutoff has already been described in the literature, we have categorized immune cell densities as high or low according to a cutoff value set at the median of each index.

**Fig. 1:**
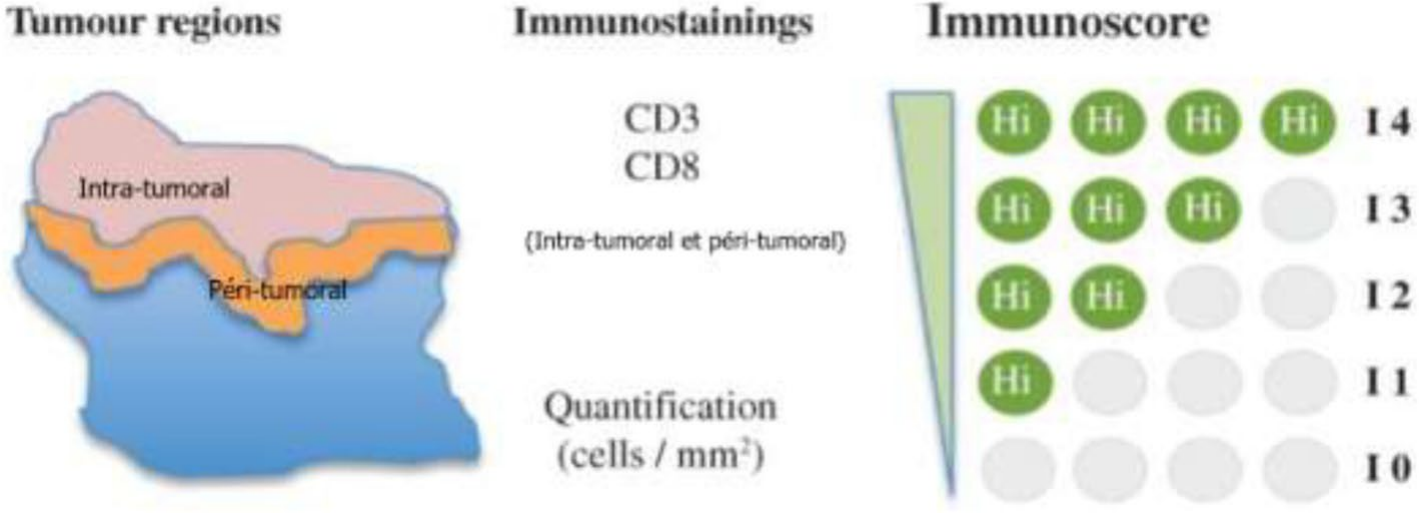
IS groups

Finally, the densities were translated into an IS by calculating the ratio CD8+/CD3+ for each case (Fig. 1). The IS was variable from I0 to I4 and was assessed as low (I0–I1) or high (I2–I3–I4) (Table 1).

**Table 1:** Characteristics of immunoscore groups

**Immunoscore**	**Characteristics**
**Immunoscore 0 (I0)**	One low rate of CD3+ and CD8+ lymphocytes (TM and IT)
**Immunoscore 1 (I1)**	One high rate of CD3+ or CD8+ lymphocytes (in either TM or IT)
**Immunoscore 2 (I2)**	Two high rates of CD3+ and CD8+ lymphocytes in three studied areas (TM or IT)
**Immunoscore 3 (I3)**	Three high rates of CD3+ or CD8+ lymphocytes (TM and IT)
**Immunoscore 4 (I4)**	Diffuse high rate of CD3+ and CD8+ lymphocytes (TM and IT)

### Statistical analysis

#### Sample size calculation

The author’s have calculated the minimal sample size using the following equation on R software (sample size for mean estimate):

$$n = [((Z^2 \alpha ):2) \times S^2]/i^2$$

n: Estimated sample size

Z^2^α = 1.96 the critical value for normal deviates for an alpha error of % that corresponds to the confidence level 95%

S: Standard deviation 5.4

i: Precision at 2

The statistical analyses were performed using SPSS software (SPSS standard version 26). The percentages were calculated for the qualitative variables and means and standard deviations for the numerical variables.

To determine the association between the IS and the clinicopathologic features of the patients, Pearson’s chi-square test and Fisher exact test were used. For survival analysis, the BC patients were subjected to Kaplan–Meier analysis. The log-rank test was used to compare the different survival curves. Multivariate survival analysis was performed on parameters with a *p*-value < 0.2 on univariate analysis using the Cox regression model. All *p*-values lower than ≤ 0.05 were considered statistically significant.

## Results

### Patient’s characteristics

Thirty of 35 patients diagnosed with invasive UC between 2011 and 2021 were included. A total of 7/30 patients have previously received chemotherapy and/or BCG therapy. Clinical and pathological findings are summarized in Table 2.

**Table 2:** Baseline characteristics of the patients

**Variable**	**Categories**	**Patients (*n* = 30)** **Number**
**Sex**	Male	27 (90%) 3(10%) 15 (50%) 15 (50%) 8 (27%) 8 (27%) 14 (46%) 19 (63%) 10 (33%) 1 (4%)
	Female	
**Age**	≤ 65 ans	
	> 65	
**Tumor size**	≤ 4.5 cm	
	> 4.5 cm	
	-	
**Multifocal tumor**	-	
	+	
	Not specified	
**Recurrence/tumor progression**	**+**	7 (23%)
**Distant metastasis**	**+**	9 (30%)
**Death**	**+**	15 (50%)
**Histological variant**	Common UC	19 (63%) 11 (37%) 25 (83%) 5 (17%) 13 (43%) 17 (57%) 9 (30%) 21 (70%) 12 (40%) 13(43,3%) 5 (16.6%)
	Other variant	
**In situ carcinoma**	Absence	
	Presence	
**Vascular invasion**	Absence	
	Presence	
**pT**	pT1	
	≥ pT2	
**Lymph node statuts**	-	
	+	
	Unspecified	

**Fig. 2:**
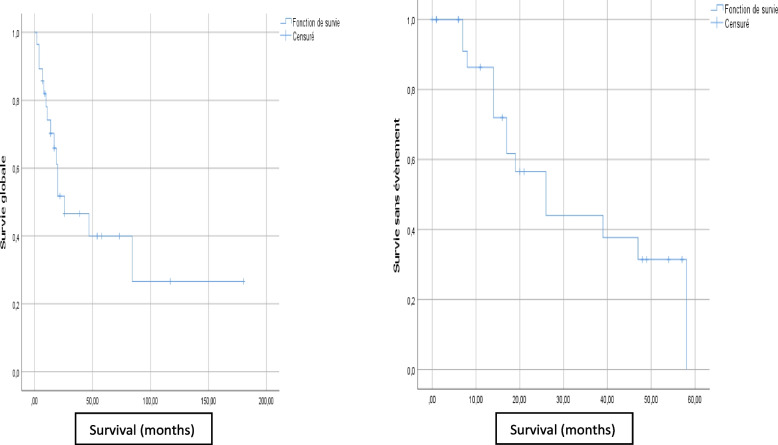
OS and DFS curves

During the follow-up, 50% of patients died. The overall survival (OS) varied from 2 to 180 months (median 18 months). The OS was 50% at 2 years and 40% at 5 years. The disease-free survival (DFS) varied from 0 to 26 months (median 26 months). DFS was 56% at 2 years (Fig. 2).

### *Immunohistochemical findings (*Figs. 3 and 4*)*

**Fig. 3:**
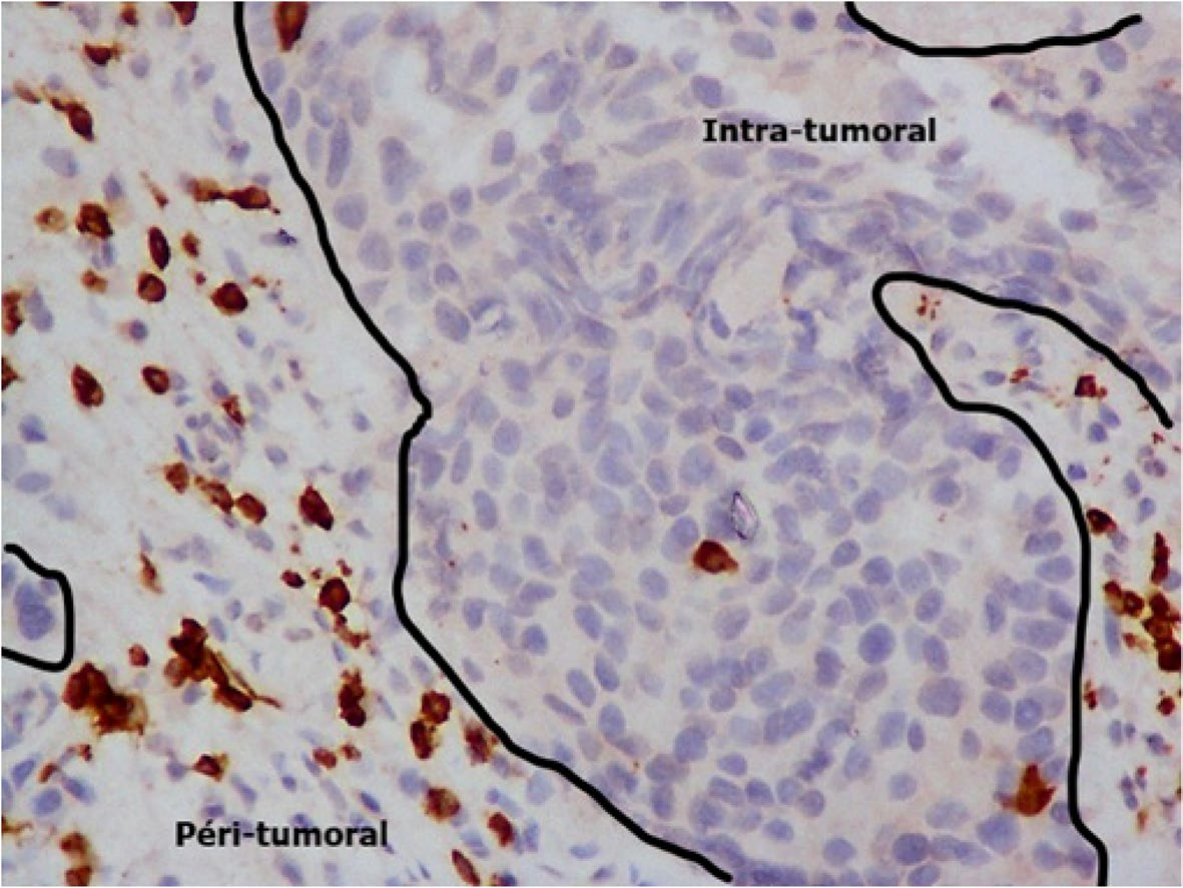
Representative image showing high density of CD3+ in PT and a low density in IT. Original magnification: ×400

**Fig. 4:**
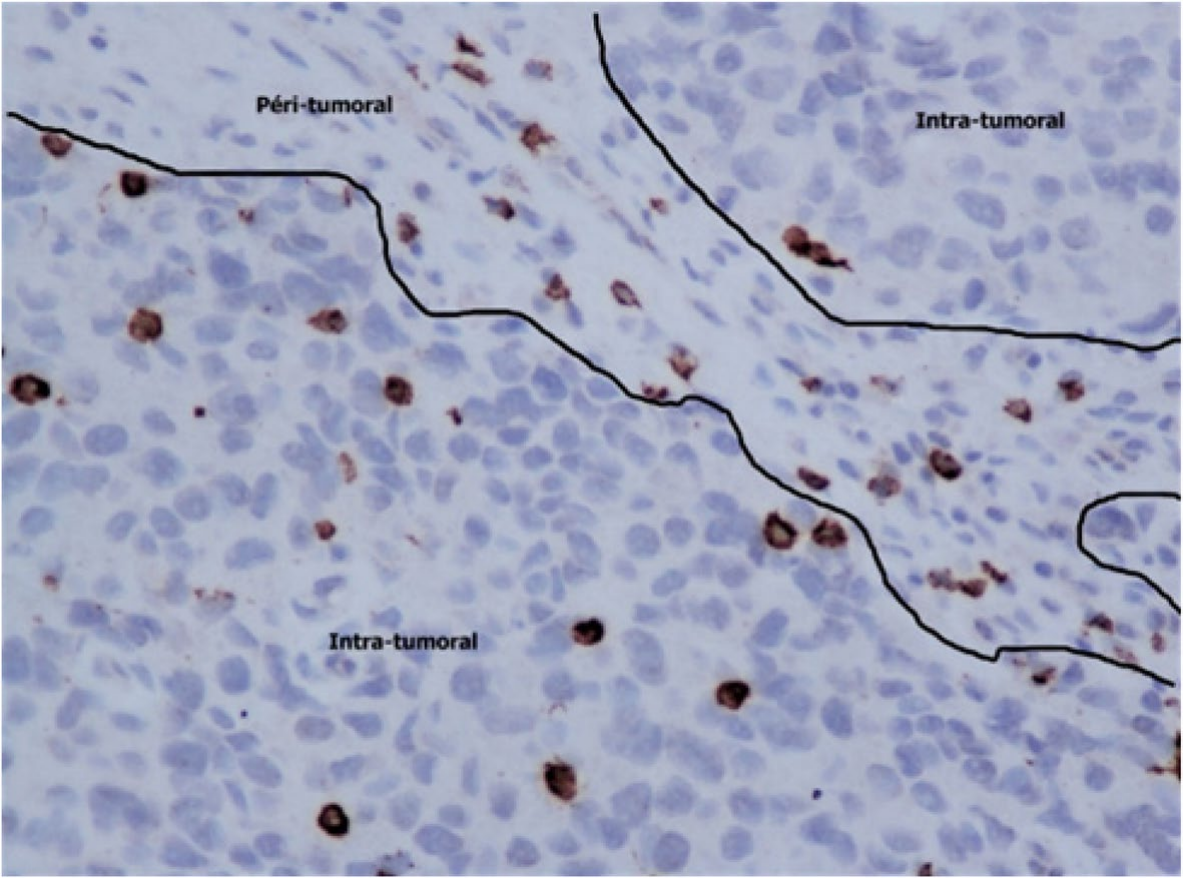
Representative image showing low density of CD8+ in IT and PT. Original magnification: ×400

#### TILs density and distribution and IS

On IT areas, the number of lymphocytes varied from 2 to 136 cells/mm^2^ for CD3+ cells and from 1 to 126 cells/mm^2^ for CD8+ cells.

On TM areas, the number of lymphocytes varied from 6 to 160 cells/mm^2^ for CD3+ cells and from 1 to 26 cells/mm^2^ for CD8+ cells (Table 3).

**Table 3:** The rates of CD3+ and CD8+ TILs on peri-tumoral and intra-tumoral

**Variable**		**Min-max (cells/mm**^**2**^**)**	**Mean (cells/mm**^**2**^**)**	**Median (cells/mm**^**2**^**)**	**Category**	**Number (%)**
**CD3+ T cells**	IT	[2–136]	27	14	Low	16 (53%)
					High	14 (47%)
	TM	[6–160]	60	56	Low	15 (50%)
					High	15 (50%)
**CD8+ T cells**	IT	[1–126]	22	12	Low	16 (53%)
					High	14 (47%)
	TM	[1–26]	36	28	Low	17 (57%)
					High	13 (13%)

The immunoscore (IS) was classified as follows:

- I0 in 6 cases (20%)
- I1 in 8 cases (27%) IS low (47%)
- I2 in 6cases (20%)
- I3 in 4 cases (13%) IS high (53%)
- I4 in 6 cases (20%)

### Univariate analysis

A statistically significant difference was found between the densities of CD3+ and CD8+ TILS on IT and TM areas (*p = 0.003 and p = 0.004*).

A low density of CD8+ TILs were associated with advanced age (*p* = 0.05), common histology of UC (*p* = 0.05), and the presence of vascular invasion (*p* = 0.02).

No significant association was found between CD3+ and CD8+ TILS and the other clinical, pathological, and survival data (Table 4).

**Table 4:** Association of CD3+ and CD8+ TILs to histological parameters using the *χ*^2^ Pearson test

**Factor**		**T CD3+ lymphocytes (IT/TM)**		***p***	**T CD8+ lymphocytes (IT/TM**		***p***
		Low	High		Low	High	
**Histological subtype**	Common (*n* = 19)	6	13	0.7		9 10	**0.05**
	Variant (*n* = 11)	5	6			1 10	
**In situ carcinoma**	Absence (*n* = 25)	11	14	0.1	8 17		0.6
	Presence (*n* = 5)	0	5		2 3		
**Vascular invasion**	Absence (*n* = 13)	3	10	0.3		1 12	**0.02**
	Presence (*n* = 17)	8	9			9 8	
**Stage pT**	pT1 (*n* = 9)	4 5		0.7		4 5	0.4
	≥ pT2 (*n* = 21)	7 14				6 15	
**Lymph node extension**	No (*n* = 12)	6	6	0.2		5 7	0.4
	Yes (*n* = 13)	3	10			3 10	

The analysis of the association of IS to clinical and pathological parameters showed a statistically significant relation between the IS and vascular invasion (*p* = 0.04). No statistically significant association was found with the other studied factors (Table 5).

**Table 5:** Association between IS and clinicopathological characteristics

**Variable**		**Immunoscore**		**p**
		Low	High	
**Gender**	Male	12	15	0.6
	Female	2	1	
**Age**	≤ 65 years	5	10	0.3
	> 65 years	9	6	
**Tumor size**	≤ 4.5 cm	4	4	1
	> 4.5 cm	3	5	
**Tumor focality**	No	9	10	1
	Yes	4	6	
**Histological subtype type**	Classic UC	10	9	0.5
	Variant	4	7	
**In situ carcinoma**	No	12	13	1
	Yes	2	3	
**Vascular invasion**	No	2	11	**0.004**
	Yes	12	5	
**pT stage**	pT1	5	4	0.7
	≥ pT2	9	12	
**Lymph node status**	No	8	4	0.1
	Yes	4	9	
**Recurrence**	No	10	11	1
	Yes	3	4	
**Metastasis**	No Yes	7 6	12 3	0.2

The univariate analysis showed that TILs and immunoscore did not impact the overall survival (OS) or disease-free survival (DFS). The OS and DFS medians were slightly superior in highly T (CD3+/CD8+)-cell infiltrated tumors as well as tumors with a high IS (Table 6).

**Table 6:** The association of TILs and IS to OS and DFS OS and DFS (univariate analysis)

**Factor**	**Category**	**OS median (CI à 95%)**	***p***	**DFS median (CI à 95%)**	***P***
**CD3+ intra-tumoral TILs**	Low	20 (18.5–21.4)	0.8	19 (0–42)	0.6
	**High**	**47 (6.7–87.3)**		**26 (8.6–43.3)**	
**CD3+ peri-tumoral TILs**	Low	20 (8.1–31.8)	0.5	17 (12.5–21.4)	0.5
	**High**	**47 (0–107)**		**39 (7.3–70.6)**	
**CD8+ intra-tumoral TILs**	Low	47 (0–117.4)	0.3	39 (10.2–67.8)	0.2
	High	17 (4–30)		14 (7–21)	
**CD8+ peri-tumoral TILs**	Low	20 (18.3–21.6)	0.3	26 (13–38.4)	0.7
	**High**	**47 (-)**		26 (0–58.7)	
**IS**	Low	20 (17.2–22.7)	0.6	19 (14–24)	0.7
	**High**	**47 (1–93.3)**		**26 (7–45)**	

*CI* confidence interval. **-**Not obtained, no sufficient

### Multivariate analysis

No statistically significant association was found between TILS densities/immunoscore and the studied clinical and pathological data. CD3+ and CD8+ TILs densities and IS did not impact overall survival and disease-free survival.

## Discussion

In the present study, an IHC-based quantification of TILs was performed. TILs were present in all cases, but we observed that the level of TILs infiltration was considerably variable among patients and even between intra-tumoral and tumor margin areas in the same patient. Indeed, high rates of CD3+ and CD8+ were more likely to be found at the tumor margin with a statistically significant difference (*p* = 0.03; *p* = 0.04).

These findings may be in part due to the patient and tumor heterogeneity and the variable molecular subtypes [11]. In fact, in a previous whole-genome gene expression analysis of UC, Sjödahl G. et al. concluded that molecular subtypes of UC display immunological responses at different levels to such an extent that a separate group of “infiltrated/immunogenic” tumors could be observed [12].

Moreover, it could be argued that the protocol for TILs quantification may considerably affect the results. On breast cancer, based on the recommendation of the consensus working group for TILs assessment, quantification is made on hematoxylin-eosin slides, and there is no need for IHC profiling [6]. However, most studies have found that only quantification of stromal TILs is reproducible, and that intra-tumoral TILs are difficult to detect on hematoxylin-eosin-stained slides and are mostly confused with tumor cell apoptosis [6]. In bladder cancer, although some studies have reported important roles for TILs, no convenient and effective counting system is in place. According to some authors, TILs quantification based on the validated recommendations of the consensus working group can be easily performed on UC. However, it is recommended to separately report stromal and intra-tumoral TILs [13]. However, as described in the present paper, most published studies that investigated on TILs in BC used the immunohistochemistry method which has many benefits comparing to the standard morphological method. Indeed, firstly, it could accurately detect the intra-tumoral immune cells component which contributes to TILs [8]. Secondly, with IHC analysis, the different subtypes of TILs are identified which seems to be of great importance since previous studies have advanced that the prognostic impact of TLS depends on the subtypes of the immune cells [14–17]. Thirdly, immunohistochemical profiling of TILs could be translated into an immunoscore (IS). The latter is a standardized scoring system derived from a measure of CD3+ and CD8+ cell densities at the tumor center and invasive margin. Findings have shown it to be a reliable and statistically significant prognostic tool for predicting the survival of patients with digestive cancers [7, 18].

In BC, Xiang-Dong Li et al. suggested that the immunoscore is also a reliable indicator of an improved prognosis for patients with UC [8]. In the same context, in their reported paper, Mlecnik B. et al. observed that high immunoscore and high density of CD8+ cytotoxic TILs correlated with a decreased risk of metastasis [5].

In the present study, the author’s results were consistent with the previous reported data. Indeed, we noted that a high IS and high levels of CD8+ TILs were significantly associated with good prognostic factors, such as the absence of vascular invasion. We also observed that the CD8+ density was inversely correlated to the age of patients, which is probably due to the aging immune system as stated by Giefing-Kroll C. et al. [19].

While other studies that investigated the prognostic role of TILs in BC suggested that high levels of CD3+ and CD8+ TILs were correlated with a poor outcome, relapse, and reduced overall survival [9, 20]. These inconsistencies may be in part justified by the different samples selection and the variable methods of TILs quantification.

In the present study, the author’s focused on the correlation of TILs and IS to clinical and pathological factors. As previously described, we found that low densities of CD8+ TILs were associated with and advanced age, with a common histological subtype and the presence of vascular invasion. Hence, at the opposite increased, CD8+ lymphocytes seem to reduce tumor extension beyond vessels and protect from vascular micrometastasis. However, we did not establish any significant correlation between TILs populations, IS, and the studied clinicopathological factors. These results were consistent with most published studies [21–24]. However, some other authors reported a positive association between TILs densities and factors such as histology variants, tumor size, grade, multiplicity, and lymph node status [14, 15, 25–27].

In BC, the prognostic impact of TILs and IS on survival is still an object of debate. Sharma et al. were the first to investigate the prognostic impact of the immune system in BC and concluded to a positive association between TILs densities and a prolonged OS and DFS on univariate and multivariate analyses [21]. Otto et al. reported a correlation between a high intra-tumoral TILs density and a prolonged OS [28]. However, based on their findings, Hulsen et al. stated that a superior OS is associated with a high peri-tumoral CD3+ density, but a high IT TILs density was associated with a deteriorated OS [9].

In the present study, no significant association between TILs densities IS and neither OS nor DFS was noted. Nevertheless, the OS and DFS medians were superior in highly T-cell-infiltrated tumors along with tumors with a high IS.

These discrepancies between the different studies regarding the prognostic value of TILs and IS on BC and its impact on survival may also be in part explained by the differences in the degree of TILs activation depending on the areas of their distribution. Indeed, it has been proven that intra-tumoral CD8+ TILs are mostly effector memory cells that are retained on the tumor site using the integrin CD103 [29]. The latter is also involved in the anti-tumoral cytotoxic activity of CD8+ TILs which could explain their association with a good prognosis. On the opposite, the TILs in the peri-tumoral area could contain a proportion of non-effector CD8+ TILs which could be related to T-cell exhaustion due to a dysfunction of the PD-1/programmed cell death ligand (PD-L1) pathway [15]. Hence, an increased rate of CD8+ PDL1 TILs in the peri-tumor areas is more likely to be associated with a bad outcome [15]. In this context, the analysis of TILs in association with the expression of PDL-1 could not only give further prognostic biomarkers but also predict the tumor response to immunotherapy.

Hence, the immune microenvironment of UC can be considered an interesting emerging biomarker for prognosis, or even as markers for bladder cancer therapy, which can be a focus of immunotherapy for bladder cancer.

However, further multicenter studies through standardized quantification methods of TILs and IS in BC are needed to definitively assess their prognostic value.

## Conclusion

The present study data revealed that the levels of TILs were significantly variable between intra-tumoral and the tumor margin areas which raise the need to standardize the quantification of these immune cells in BC.

Despite the small cohort size and use of retrospective material in this study, our findings strengthen the potential prognostic utility of TILs and IS in UC of the bladder. Hence, further investigations on larger populations are highly recommended, and consensus recommendations on TILs assessment and scoring should be achieved to standardize the use of these potential prognostic markers among pathologists and clinicians.

## Data Availability

Data sharing is not applicable to this article as no datasets were generated or analyzed during the current study.

## Abbreviations

BC: Bladder cancer
UC: Urothelial carcinoma
RC: Radical cystectomy
TILs: Tumor-infiltrating lymphocytes
IHC: Immunohistochemistry
IS: Immunoscore
TM: Tumor margin
IT: Intra-tumoral
BCG: Bacillus-Calmette-Guerin
OS: Overall survival
DFS: Disease-free survival
